# Disruptive Innovation: Implementation of Electronic Consultations in a Veterans Affairs Health Care System

**DOI:** 10.2196/medinform.4801

**Published:** 2016-02-12

**Authors:** Gouri Gupte, Varsha Vimalananda, Steven R Simon, Katerina DeVito, Justice Clark, Jay D Orlander

**Affiliations:** ^1^ School of Public Health Department of Health Policy and Management Boston University Boston, MA United States; ^2^ Center for Healthcare Organization and Implementation Research (CHOIR) Edith Nourse Rogers Memorial Veterans Affairs Medical Center Bedford, MA United States; ^3^ Section of Endocrinology, Diabetes and Metabolism School of Medicine Boston University Boston, MA United States; ^4^ VA Boston Healthcare System Center for Healthcare Organization and Implementation Research (CHOIR) Boston, MA United States; ^5^ VA Boston Healthcare System Section of General Internal Medicine Boston, MA United States; ^6^ Brigham and Women’s Hospital Division of General Internal Medicine and Primary Care Boston, MA United States; ^7^ Harvard Medical School Boston, MA United States; ^8^ Harvard School of Public Health Department of Nutrition Boston, MA United States; ^9^ School of Medicine Section of General Internal Medicine Boston University Boston, MA United States

**Keywords:** remote consultations, clinical communication, electronic consultation, telehealth, clinical information, decision making, telemonitoring, eHealth infrastructures

## Abstract

**Background:**

Electronic consultations (e-consults) offer rapid access to specialist input without the need for a patient visit. E-consult implementation began in 2011 at VA Boston Healthcare System (VABHS). By early 2013, e-consults were available for all clinical services. In this implementation, the requesting clinician selects the desired consultation within the electronic health record (EHR) ordering menu, which creates an electronic form that is pre-populated with patient demographic information and allows free-text entry of the reason for consult. This triggers a message to the requesting clinician and requested specialty, thereby enabling bidirectional clinician-clinician communication.

**Objective:**

The aim of this study is to examine the utilization of e-consults in a large Veterans Affairs (VA) health care system.

**Methods:**

Data from the electronic health record was used to measure frequency of e-consult use by provider type (physician or nurse practitioner (NP) and/or physician assistant), and by the requesting and responding specialty from January 2012 to December 2013. We conducted chart reviews for a purposive sample of e-consults and semi-structured interviews with a purposive sample of clinicians and hospital leaders to better characterize the process, challenges, and usability of e-consults.

**Results:**

A total of 7097 e-consults were identified, 1998 from 2012 and 5099 from 2013. More than one quarter (27.56%, 1956/7097) of the e-consult requests originated from VA facilities in New England other than VABHS and were excluded from subsequent analysis. Within the VABHS e-consults (72.44%, 5141/7097), variability in frequency and use of e-consults across provider types and specialties was found. A total of 64 NPs requested 2407 e-consults (median 12.5, range 1-415). In contrast, 448 physicians (including residents and fellows) requested 2349 e-consults (median 2, range 1-116). More than one third (37.35%, 1920/5141) of e-consults were sent from primary care to specialists. While most e-consults reflected a request for specialist input to a generalist’s question in diagnosis or management in the ambulatory setting, we identified creative uses of e-consults, including requests for face-to-face appointments and documentation of pre-operative chart reviews; moreover, 7.00% (360/5141) of the e-consults originated from our sub-acute and chronic care inpatient units. In interviews, requesting providers reported high utility and usability. Specialists recognized the value of e-consults but expressed concerns about additional workload.

**Conclusions:**

The e-consult mechanism is frequently utilized for its initial intended purpose. It has also been adopted for unexpected clinical and administrative uses, developing into a “disruptive innovation” and highlighting existing gaps in mechanisms for provider communication. Further investigation is needed to characterize optimal utilization of e-consults within specialty and the medical center, and what features of the e-consult program, other than volume, represent valid measures of access and quality care.

## Introduction

Electronic consultations (e-consults) can be broadly defined as a platform for provider-provider consultation facilitated by health information technology such as an electronic health record (EHR) or Web-based portal. E-consults are most frequently used by primary care providers (PCPs) to access specialist input on clinical questions that can be addressed through chart review, thereby avoiding the need for a patient visit to the specialty clinic [1-3]. Generally, the intent of e-consults is to improve efficiency and overall quality of care by increasing access to specialty input while reducing unnecessary face-to-face visits [4-8].

While e-consults are a promising innovation, adoption is not widespread [3]. However, with health care reform in the United States and beyond [9], models of integrated care delivery may incentivize institutions to develop e-consult programs to increase access to specialty care while limiting costs [10]. Successful implementation will require a better understanding of how e-consults are being used and the reasons for those uses among both requesters, who are typically PCPs, and responders, who are typically specialists [3,4,11]. Ultimately, patients, clinicians, and policy-makers will need to know how e-consults influence the quality, safety, and cost of health care.

E-consults are a key component of the US Veterans Affairs (VA) health care system’s efforts to improve access to specialty care for Veterans, many of whom need to travel long distances to see their providers [8,12-16]. To gain insight into the ongoing rollout and implementation of e-consults across VA, we studied e-consult usage in a large VA health care system. We used quantitative and qualitative methods to characterize the various ways e-consults are used and to explore clinicians’ and hospital leaders’ attitudes and motivations underlying the patterns observed.

## Methods

### Study Design

An observational quality improvement study using mixed-methods was conducted. We extracted data from the EHR to quantify e-consult usage over time, by provider type (physician or nurse practitioner (NP) and/or physician assistant), and by specialty. We then used qualitative methods to explore both common and unexpected uses of e-consults. Our study utilized the mixed-methods sequential explanatory design described by Creswell and others [17,18]. This design involves collecting and analyzing first quantitative and then qualitative data in two consecutive phases within one study. The second phase builds on the first, in that the qualitative data and analysis help to explain the quantitative results obtained earlier. In our study, we conducted quantitative data collection and analysis to describe the patterns of e-consults use. We identified wide variation in uptake among users, as well as several unexpected patterns of use between and within services. We then used two qualitative methods to help explain our quantitative findings. We purposefully selected both high and low e-consult users as well as administrators, and interviewed them to better understand the reasons for variation in uptake across individuals, and to understand reasons for unexpected uses.

The primary analysis was conducted by three team members (GG, VV, and JC) and was reviewed with the entire study team. This analysis by the project team was cycled back and forth between individual cases and comparisons across cases to capture evolving themes and to understand the dynamics among users of e-consults. Throughout this process, the project team revisited the full interview notes for more detailed analysis on points of interest and to pursue hypotheses.

### Setting

VA Boston Healthcare System (VABHS) is a tertiary care system consisting of three main campuses and five community-based outpatient clinics. VABHS provides primary care through a patient-centered medical home model to over 32,000 Veterans with more than 750,000 outpatient visits annually. The main campuses provide acute inpatient care, transitional, palliative, hospice, and nursing-home levels of care, as well as an extensive portfolio of specialized ambulatory procedures and consultative specialty clinics. VABHS is an academic center with multiple affiliations with health care training institutions, hosting hundreds of students, residents, and fellows each year. VABHS is the main referral center for five New England states.

### Implementation of e-Consults at VABHS

In January 2011, e-consults were launched at VABHS for selected medical specialties [8,15]. The Department of Medicine promoted use through discussions with primary care leadership and emails to PCPs describing the availability and purpose of e-consults. By early 2013, e-consults had expanded to all clinical services, including all surgical specialties, mental health, and pharmacy with the exception of radiology. Teledermatology was not included as it has been used at VABHS for utilizing collection, storing, and forwarding of new images, and is considered another type of health information technology application. Clinicians are able to request an e-consult in the same way they request a face-to-face consultation, by requesting the service from a menu within the EHR. Certain specialties also accept e-consults from other VA facilities in New England.

Although e-consults in most health care systems are intended for PCPs to request specialty consultations [3,10,19], the VA platform allows any provider with ordering privileges to request an e-consult [20]. The clinician selects the desired consultation within the EHR ordering menu, which creates an electronic form that is pre-populated with patient demographic information and allows free-text entry of the reason for consult. The EHR has a built-in function that allows staff and clinicians to add comments in a separate free-text field within the consultation request. This triggers a message to the requesting clinician and requested specialty, thereby enabling bidirectional clinician-clinician communication. Each specialty has the ability to tailor the e-consult form to solicit or require specific information elements from the ordering clinician. For example, some surgical specialties require the ordering clinician to indicate whether the patient is taking anti-coagulant medications, which would need to be considered before a surgical procedure. Once the ordering clinician completes and electronically signs the request for consultation, it is delivered electronically to the requested specialty. Each specialty routes the incoming consultation requests according to its own preferences. For example, some specialties have a clerk perform an initial review of all consultation requests before forwarding them to individual specialist physicians, while others have one or more clinicians receiving all the consultation requests directly from the ordering clinicians. Consultants can choose to convert an e-consult to a request for a face-to-face visit, or vice versa [21]. Responding providers receive workload credit, a measurement of clinician work that is used within VA for resource allocation, based upon relative value units for each e-consult completed. In February 2014, VA approved the allocation of workload credit to e-consults based on self-reported time spent completing the e-consult in three discrete ranges (1) less than 15 minutes; (2) 15-30 minutes; and (3) greater than 30 minutes. These quantities correspond to so-called levels 2, 3, and 4 for outpatient visit complexity. Responding providers are expected to answer e-consults within three working days.

### Data Collection and Analysis

We used the VA EHR to extract quantitative data from January 2012 to December 2013. Data included information on the sending and receiving provider type and specialty, and the date and time of actions on each e-consult. All specialties except radiology participated in the usage of e-consults at the point of the data collection. The date and time stamps allowed us to calculate time to completion, measured as the elapsed time between the signature of the requesting clinician on the e-consult request and the signature of the clinician providing consultation on the completed e-consult form. We used findings from the quantitative data to inform 30-minute, semi-structured interviews with frequent and less frequent requesters and responders and both clinical specialty and hospital leadership in order to better understand e-consult utilization as well as barriers and facilitators of use. We contacted and interviewed 17 medical doctors (MDs), 9 NPs, 1 doctor of pharmacy, and 4 hospital leaders (including 2 chiefs of specialties) for a total of 31 providers from 21 specialties. All individuals we contacted agreed to participate. Using a semi-structured interview guide, we asked providers about their knowledge, usage, experience, and feedback related to e-consults. We asked leadership about the strategies used for promoting e-consult implementation and uptake, assessment methods, and future plans. Interviews were audio recorded and transcribed verbatim for analysis. We held team meetings to identify thematic categories related to our project goals, refine their meaning, discuss alternative interpretations, and reach an agreement on representative quotations for each category.

This work was reviewed by the VABHS Institutional Review Board and was determined to be quality improvement rather than human subjects’ research.

## Results

### Quantitative Findings

Our dataset contained information on a total of 7097 e-consults, representing all VABHS e-consults during the study period. In 2012, 1998 (28.15%, 1998/7097) e-consults were completed, compared with 5099 (71.85%, 5099/7097) in 2013, representing a 150% year-to-year increase. More than one quarter (27.56%, 1956/7097) of the e-consult requests originated from VA facilities in New England other than VABHS. Further analyses were limited to the 5141 e-consults originating within VABHS.

The distribution of clinical locations from where e-consults originated within VABHS are displayed in Table 1. More than one-third of the requests for e-consults (37.35%, 1920/5141) originated in primary care, representing the single largest requesting specialty. Nearly one-third (32.0%, 1645/5141) of the e-consults were directed to general surgery and surgical subspecialties.

**Table 1:** Distribution of e-consults within VABHS by location from where the consult originates from January 1, 2012 to December 31, 2013 (N=5141).

Specialties		Number of e-consults sent, n (%)
**Medicine**		2318 (45.09)
	Primary care	1920 (37.35)
	Other medical sub-specialties^a^	398 (7.74)
**Surgery**		1757 (34.18)
	Orthopedics	634 (12.33)
	Pre-admission testing clinic	370 (7.20)
	General surgery	211 (4.10)
	Other surgical sub-specialties^b^	542 (10.54)
**Other**		1066 (20.74)
	Sub-acute chronic and inpatient care	365 (7.10)
	Medical acute care	136 (2.65)
	Mental health/psychiatry	75 (1.46)
	Other sub-specialties^c^	490 (9.53)

^a^Medical sub-specialties include pulmonary (2.12%, 109/5141), cardiology (1.44%, 74/5141), gastroenterology (1.09%, 56/5141), geriatrics (0.70%, 36/5141), renal (0.58%, 30/5141), rheumatology (0.49%, 25/5141), oncology (0.35%, 18/5141), sleep (0.31%, 16/5141), post-discharge clinic (0.23%, 12/5141), hematology (0.18%, 9/5141), infectious disease (0.12%, 6/5141), dermatology (0.06%, 3/5141), allergy (0.04%, 2/5141), endocrinology (0.02%, 1/5141), and palliative care (0.02%, 1/5141).

^b^Surgical sub-specialties include optometry (2.49%, 128/5141), ear nose and throat (2.22%, 114/5141), urology (1.50%, 77/5141), ophthalmology (1.09%, 56/5141), thoracic (0.97%, 50/5141), vascular (0.41%, 21/5141), gynecology (0.31%, 16/5141), podiatry (0.21%, 11/5141), bariatric (0.11%, 6/5141), cardiac surgery (0.08%, 4/5141), and plastic surgery (0.06%, 3/5141).

^c^Other includes administrative (2.82%, 145/5141), undefined (1.24%, 64/5141), neurology (1.19%, 61/5141), urgent care (1.46%, 75/5141), spinal cord injury (1.09%, 56/5141), radiology (0.78%, 40/5141), pharmacy (0.62%, 32/5141), surgical acute care (0.49%, 25/5141), rehab medicine (0.31%, 16/5141), anesthesia (0.19%, 10/5141), occupational health (0.14%, 7/5141), audiology (0.08%, 4/5141), dental (0.12%, 6/5141), nutrition (0.04%, 2/5141), prosthetics (0.04%, 2/5141), and radiation therapy (0.02%, 1/5141).

The median time to completion across specialties was 2.2 working days (range 0.8-56). A subset of e-consults (45.77%, 2353/5141) included consultants’ indication of time spent completing the e-consult. Most (83.00%, 1953/2353) indicated that they spent less than 15 minutes, while 11.00% (259/2353) spent 15-30 minutes and 5.00% (141/2353) spent more than 30 minutes completing the e-consult. We identified variability in the number of e-consults requested by individual clinicians. A total of 64 NPs requested 2407 e-consults (median 12.5, range 1-415). In contrast, 448 physicians (including residents and fellows) requested 2349 e-consults (median 2, range 1-116). A total of 385 e-consults, representing 7.49% (385/5141) of all e-consults during the study period, were submitted by staff members other than NPs and physicians. Department of Medicine physicians requested a median of 2.0 e-consults (range 1-103), while NPs in Medicine requested a median of 5.0 e-consults (range 1-88).

**Figure 1:**
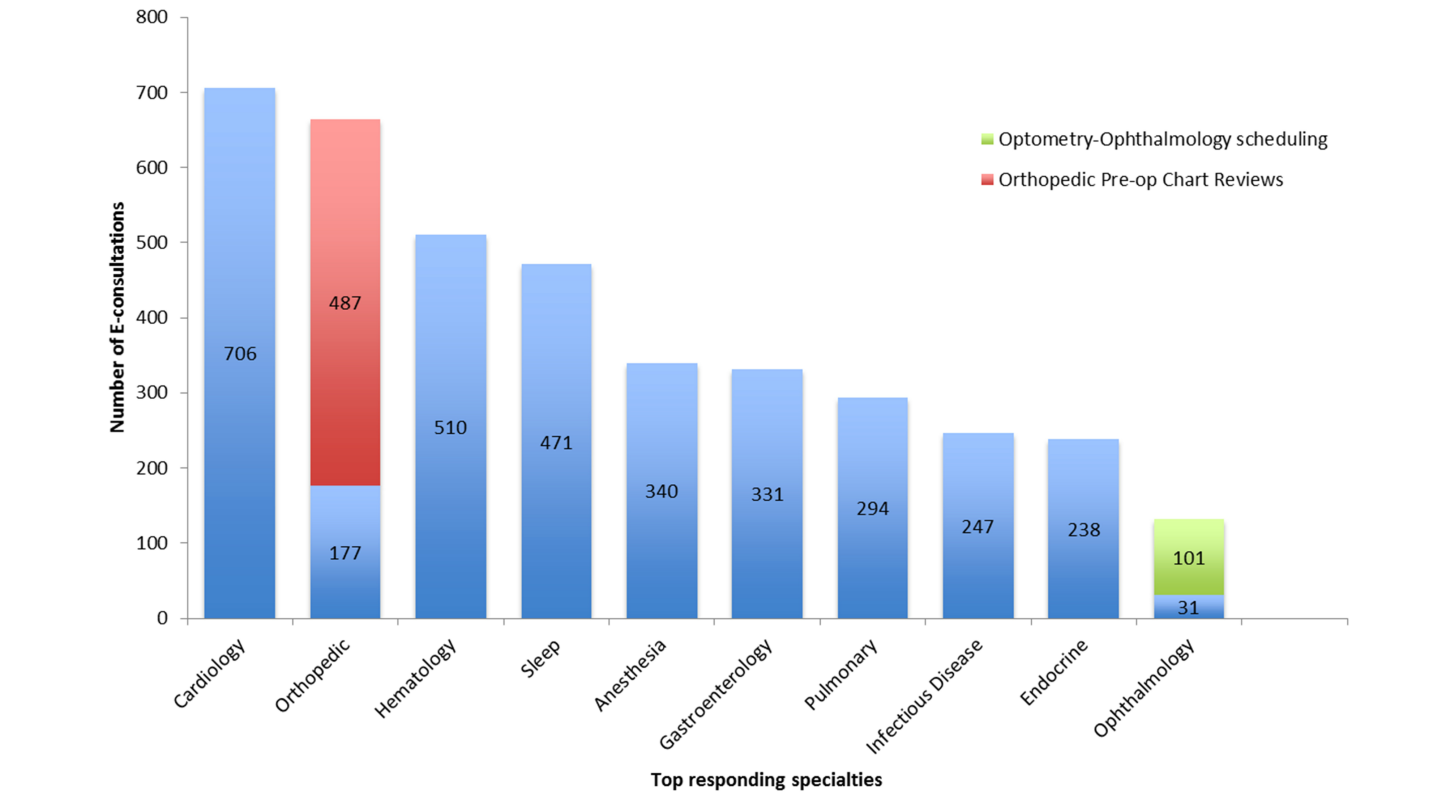
Distribution of e-consults within VABHS by specialties receiving the most e-consult requests from January 1, 2012 to December 31, 2013 (N=5141).

The distribution of specialties at VABHS receiving the most e-consults requests is displayed in Figure 1. The medical specialties consulted most frequently were cardiology (13.73%, 706/5141), hematology (9.92%, 510/5141), sleep medicine (9.16%, 471/5141), gastroenterology (6.44%, 331/5141), and pulmonary (5.72%, 294/5141). Frequently consulted specialties within surgery included orthopedics (12.92%, 664 /5141) and ophthalmology (2.57%, 132/5141). We unexpectedly identified a number of e-consults submitted within one specialty. For example, 27.72% (487/1757) of e-consults to surgery and surgical specialties were submitted by a clinician in orthopedics to the orthopedics service (ie, an intra-specialty e-consult). These intra-specialty e-consults to orthopedics accounted for 9.47% (487/5141) of all e-consults within the VABHS (see Figure 1). Similarly, 5.74% (101/1757) of e-consults to surgery and surgical specialties were submitted by optometry to ophthalmology, while 1.76% (31/1757) of e-consults to surgery and surgical specialties were submitted by ophthalmology to optometry.

### Qualitative Findings

#### Unanticipated Uses of e-Consults

The flexibility of the e-consult mechanism was often considered an advantage by specialists, as each specialty could develop its own triage and response mechanism. This flexibility also allowed adaption of the e-consult mechanism for several uses that were not originally intended. Quantitative data had revealed a number of e-consults submitted within the same specialty, or within two divisions of the same specialty. For example, we identified numerous e-consults requested from optometry to ophthalmology and vice-versa. Upon inspection of their content and interviewing providers, we found the majority of these e-consults were submitted to facilitate appointment scheduling. Some were scheduling requests within the same specialty at VABHS, while others were requests for assistance in scheduling post-ophthalmologic follow-ups at VA sites closer to patients’ homes.

We identified 588 within-specialty e-consults, particularly among surgical specialties. Through chart review and interviews we found that the majority of these within-specialty e-consults, most frequently occurring in orthopedics, but also in general surgery and other surgical specialties, had been adapted to document and facilitate pre-operative assessments. We ascertained that NPs would generate an e-consult to themselves to document a detailed pre-operative chart review. Based upon this review, NPs often solicited consultation from other specialties through additional e-consults to address issues such as pre-operative evaluation of cardiac risk (“cardiac clearance”) and pre-operative medication management (eg, anticoagulation). This work occurred prior to scheduling the patient for the surgical procedure and prior to the NP obtaining a formal history and conducting a physical examination in the pre-operative testing clinic; clinicians explained that the intention was to decrease the likelihood that scheduled elective procedures would need to be delayed. Prior to implementation of the e-consult system, NPs documented their chart review in various locations of the medical record. The e-consult system provided a uniform and easy-to-find location for such documentation that also ensured documentation of the clinical work completed (ie, workload credit). Benefit to patients was noted, as one surgical subspecialty chief said, “I think they (patients) benefit by having all their care coordinated before they come down here, too, so they don’t have to keep coming back multiple times.”

#### Benefits of e-Consults From Primary Care Providers’ Perspective

PCPs that used e-consults frequently were unanimous in the opinion that e-consults were easy to use, useful, and increased timeliness of and access to specialty care. As one provider said, “I’m a huge fan of it and a big advocate.” PCPs reported that placing e-consults was straightforward and that they were “pretty easy to find in the EHR.” The free-text format of the e-consult allowed a welcome flexibility in writing the reason for consultation with greater or lesser amounts of detail, depending on the clinical situation. The usefulness of e-consults was related to both the rapidity of the specialist response and the provision of a mechanism for asking simple clinical questions. One PCP noted, “I would define an e-consult as a higher-level question for a specialist that could be safely answered by a chart review.” Having a form of communication that was clinically oriented but asynchronous in nature seemed to empower PCPs to seek formal specialty consultation in circumstances where they previously would seek consultation informally such as via a hallway conversation, an e-mail, or phone call, often labeled curbside consultations [20]. As a provider commented, “Things that historically would have been curb-sided can now be documented as a formal conversation.” PCPs also noted that they now found themselves completing e-consult requests in situations where they previously might not have pursued specialist input due to barriers in reaching a specialist colleague and when formal referral of the patient for face-to-face visit did not seem indicated.

The easy availability of e-consults was particularly valuable for primary care NPs at community-based outpatient clinics. For these providers, the geographic distance between themselves and the hospital-based specialists generally precluded access to specialist input through informal conversations. Most PCPs perceived that e-consults resulted in fewer face-to-face consultations, more efficient medication management, expedited diagnostic testing in lieu of or in preparation for a specialty visit, and more effective communication with specialists. We also interviewed two providers who infrequently used e-consults who stated their reason for low use was because they were unfamiliar with the process itself.

#### Benefits of e-Consults From Specialists’ Perspective

Like PCPs, specialists noted a major benefit of e-consults to be fewer unnecessary face-to-face consultations. Specialists perceived more available appointment times for scheduling patients who did require face-to-face visits. As one specialist said, “It saves patients a lot of time, it makes us more efficient because we can take care of the patients who really need our services and are really sick, and, ultimately, you can probably reduce manpower too.” Specialists particularly valued having the ability to convert face-to-face consultation requests to e-consults when they deemed it appropriate.

Converting face-to-face consultation requests to e-consults was most prevalent in sleep medicine. Sleep medicine physicians, faced with extremely long wait times for clinic appointments, found that in many cases chart review was sufficient to identify which patients were at high risk for having obstructive sleep apnea, and therefore required a sleep study. Conversion of face-to-face consultation requests to e-consults allowed the sleep medicine specialist to render an opinion and order a sleep study for the patient within a few days without the need for a face-to-face evaluation. Sleep medicine physicians indicated that this approach was reported to improved clinic access and dramatically reduced time to ordering and completing a sleep study. In many cases, patients would be diagnosed and started on therapy before having their first live face-to-face consultation visit in the sleep medicine clinic. Sleep medicine physicians did question whether the quality of care and adherence to treatment would be improved if patients had been seen by them before diagnostic testing was completed and therapy initiated.

Specialists discussed the potential for e-consults to support PCP education, in that the consult questions often represented gaps in clinical knowledge that could be addressed with detailed information that might later be used as a reference. They noted that success of this strategy depended on the engagement of the referring provider in the learning process and that some clinicians made nearly identical referrals repeatedly. This pattern was observed most commonly among e-consults submitted by NPs in surgical specialties to medical subspecialties, such as hematology and cardiology, as part of a pre-operative assessment.

#### Concerns About e-Consult Implementation and Impact on Workflow

While PCPs did not report concerns about e-consults, specialists receiving the highest number of e-consults reported workflow and workload issues due to the increase in volume overtime. Specifically, they complained about the lack of a pre-implementation evaluation of e-consults’ impact on the work of the section specialty and the individual providers. E-consults originating outside of VABHS were particularly challenging and time-consuming to complete, since the mechanism for reviewing relevant information outside the main facility involves use of an associated EHR software package that is not easily searchable for specific data. Satisfaction with e-consults varied greatly between individual specialists. For example, a hematologist felt his specialty was particularly well suited to e-consultation and estimated that the hematology unit completed nearly 50% of all new requests for consultations via the e-consult process. In contrast, another individual specialist described e-consults as “an unfunded mandate” created by leadership, which was poorly integrated within the existing workload and fraught with potential legal concerns based on providing advice on a patient who was never examined in person.

One specialist reported that e-consults could contribute to breaks in continuity of care, as the respondent to an e-consult may be different from the specialist who previously saw a given patient in person. Many specialists noted that similar questions were asked repeatedly by the same providers, suggesting to them that the purpose of the e-consult for some individuals was documentation rather than clinical support and knowledge transfer. While they understood that some providers may want documentation due to a lack of clinical confidence, the e-consults for this purpose created excess and unwelcome work for some consultants.

#### Leadership Engagement to Promote e-Consult Usage

Interviews with hospital and department-level leadership revealed strong enthusiasm for e-consults. One clinical service chief noted “It allows for better triaging of consults and in theory may be improving access to subspecialty clinics.” Leaders promoted e-consults in a manner similar to how they encouraged their constituencies to embrace other information technology initiatives to improve specialty access, such as telehealth. Under this model, service-level leadership delegated staff to act as champions for e-consults across clinical services. Two hospital leaders discussed the challenges of implementing e-consults, noting “There was a lot of infrastructure building for e-consults and a lot of education that had to be done.” They described a need for more effective strategies to encourage uptake across providers and the potential benefit of incorporating structured fields with mandatory data elements in the e-consult forms to ensure that relevant information is included both by providers requesting consultation and those providing it.

Hospital leaders were not explicitly involved in measuring or tracking the use of e-consults. Use across the medical center was monitored at the regional Veterans Integrated Service Network (VISN) level only at the time of our interviews. They understood that the number of e-consults is tracked by specialty, with and considered a proxy for access to specialist expertise. VABHS hospital administrators did not have an available mechanism to provide a more detailed assessment of the content, quality or impact of e-consults on an ongoing basis.

#### Recommendations From Users of e-Consults

We closed our semi-structured interviews by asking for recommendations to improve the e-consult process. PCPs suggested that leadership should reach out to more providers, specialties, and specialists to improve buy-in of the e-consult process and increase both the number of clinicians requesting e-consults and the number of specialties providing consultation by this mechanism. Specialists suggested specialty-specific discussions to address the perception of an “unfunded mandate,” individual performance measurement, legal issues, workflow consideration, and measurement of outcome metrics by the usage of e-consults to create more awareness and engagement. One particular request made during the interviews was about clarity on how credit for work completed is assigned for consultants, as only the lowest level of workload credit was allowed when e-consults were initially launched. VA addressed this in February 2013 with nationwide approval of three different levels of workload credit based upon self-reported time spent completing the e-consult (reported in the quantitative results above). Leadership discussed the need for better evaluation strategies, which could include patient satisfaction scores, provider feedback, process measures, and qualitative workflow impacts.

## Discussion

### Principal Findings

We undertook this quality-focused evaluation to characterize the usage of e-consults in a large VA health care system, and to describe how the experience of providers and hospital leaders shapes such use. Nearly one-third of e-consults originated from clinicians outside VABHS, representing robust use of the e-consult mechanism to improve specialty access for patients at locations with limited specialty services. As expected, PCPs were the most frequent requesters of e-consults. Using qualitative methods to better understand patterns observed in our quantitative data, we identified innovative and unexpected ways that e-consults are being used to expedite evaluation of sleep disorders, for administrative communication between related specialties, and for pre-operative documentation within a specialty. Overall, both PCPs and specialists felt that e-consults improved timeliness of specialty input and reduced unnecessary face-to-face consultations. PCPs were generally highly satisfied with e-consults, while specialists had concerns related to workload and workflow.

As reported in other health care systems, we found that cardiology and hematology were frequent recipients of e-consults [19,22,23]. Further study is needed to determine whether this finding represents an overall high volume of patient need for such services, particular suitability of e-consults to these specialties, or a combination of these and other factors. The particularly high use of e-consults in sleep medicine and orthopedics was largely explained by the specific use of e-consults as adaptations to other system shortcomings; in the former, limited specialty clinic availability, and in the latter, a lack of means to document clinical work that did not include a patient visit. As noted in other studies, dermatology e-consults were not observed in the data as they present as “teledermatology” utilizing collection, storing, and forwarding of new images, and are considered another type of health information technology application [5]. Many of the e-consults to ophthalmology, the second most common recipient in surgical specialties, were requests from optometry for assistance in arranging logistically complicated post-operative plans. The adoption of e-consults for uses other than the originally intended purpose suggests that e-consults in our health care system have become a “disruptive innovation.”

Disruptive usage begins when an innovative product takes root among a group of users because it addresses previously unmet needs [24-27]. As knowledge spreads about the utility of the innovation to address these unmet needs, the innovation rapidly garners support and utilization climbs [25]. Disruptive innovations are generally convenient, easy to use, and simpler than existing or prior systems [25,27]. In addition, they are aligned and blend with a pre-existing technology infrastructure; in the case of e-consults, the existing infrastructure is the EHR. E-consults have emerged within VA as a disruptive innovation in part because the existing infrastructure and clinical processes were sufficiently flexible to tolerate the innovation. The VA EHR allows any provider with ordering privileges to request an e-consult from any other service; any type of question or request could be entered into the free-text field, without constraint to specific clinical situations or diagnoses. Hence, the availability of e-consults has led to providers asking more questions of their specialty colleagues. Furthermore, each specialty has the flexibility to develop its own mode of triage and assignment of responsibility for responding to submitted e-consults.

We found a rapid increase in e-consult use since 2011, with high levels of satisfaction among requesters. Users have actively been promoting e-consult use amongst their peers, with the encouragement of clinical leadership. These users are applying e-consults in unexpected ways to address various shortcomings with existing processes; in short, the avid users are making e-consults a disruptive innovation. One result of disruptive innovation is that it identifies previously hidden needs in the current system [24-26]. Our work raises the question of whether unexpected uses of e-consults as workarounds for system needs represent appropriate use. Unexpected and unintended uses of e-consults should be explored in other health care settings, and as e-consult use expands, each health care system will need to develop protocols and policies for managing such unanticipated use and its consequences.

High satisfaction with e-consults among PCPs has been reported in studies of e-consult use in other health care systems. Consistent with those studies, PCPs in this study appreciated the ease of access to specialists, the timeliness of specialty input, and the perception of reduced travel for patients [3,10,28]. In comparison, we identified considerable variation in satisfaction among specialists, a finding that is also consistent with prior work [4,11,20,29]. While most specialists perceived a reduction in unnecessary face-to-face visits, some were concerned about overall higher workload due to increasing e-consult volume. Addressing specialists’ concerns about the use of e-consults will be essential as this technology is expanded more broadly. The utility of e- consultation and ultimately the success of the e-consult program will depend in part on the willingness of specialists to answer appropriate e-consult requests as presented, rather than converting them to face-to-face consultations, and on the quality of the specialists’ response itself. How specialists accept and respond to e-consults are, to some degree, functions of the types of questions being asked by requesters and by the perceived benefits of e-consultation to the specialist. Our work suggests that better communication between specialists, requesting providers, and leadership about the needs and expectations of each group would foster increased uptake among specialists. While not addressed directly in our interviews, the role of the patient in e-consults merits further examination.

Concurrent with our work, some tangible improvements in the use of e-consults occurred in our institution. During our interviews, we found that one specialty was unaware of the mechanisms for identifying incomplete consults (ie, those consults that were requested but unanswered). Through other interviews, we identified problems in routing of consults to incorrect staff in another service. In both cases, we provided a brief tutorial, improving the specialty’s management of their e-consult load. We also provided feedback to our office of information systems on the menu within the EHR for selecting e-consults; this feedback led to reorganization of the types of e-consults in the menu, improving usability. While this study was not designed for rigorous evaluation of the effects of e-consults on clinical practice or outcomes. Our findings have been received with interest among primary care and specialist clinical leaders within our institution and in the regional network of VA facilities.

### Limitations

We undertook this project as a quality improvement study in a single health care system within VA. Though the specifics of e-consult use may vary across sites, PCP-to-specialist use is a common finding. Numerous specialties were represented in our data, but in most of them there were a small number of specialist physicians actually involved in triaging and responding to e-consults, limiting the generalizability of our findings. However, our mixed-methods approach of starting with quantitative data, examining de-identified e-consults from frequent users, and following up with semi-structured interviews helped us better understand use, workflow, and variability. Examining data more broadly across VA and eventually to other health care systems would provide a more robust characterization of all these issues. Another possible limitation of our analysis is that we used an administrative database, which did not include certain information about e-consults, such as whether they were converted to or from face-to-face consults, or the time to answer. Thus, our count of the numbers of e-consults may underestimate the true quantity. However, since our aim was to identify trends in e-consult use, rather than absolute quantity, we do not believe inclusion of these e-consults would meaningfully change the trends we observed. Our analyses excluded more than one-fourth of the e-consults that were submitted from outside VABHS, as full information on these was not available at the time of the study. Future evaluations should explore the content and circumstances of these consults in comparison with those submitted within VABHS.

E-consults generate a modest amount of workload credit for those responding to the request for consultation, but this workload credit is an internal metric and not linked to insurance claims or any other external monitoring system. As such, there is no built-in audit system in place to assure quality or appropriateness of use. Our findings strongly suggest that the number of e-consults is not a valid metric for access to specialty care services, such that reliance on measures of volume is likely to result in erroneous conclusions about access to care. Specific examination of the clinical content of e-consults may be necessary to determine the extent to which this innovation is actually improving Veterans’ access to high quality specialty care, a major goal of improving overall care at the VA [30].

### Conclusions

Our study shows that e-consults facilitate access to specialty expertise for PCPs, but that use across clinical services has become more widespread than originally intended. Further investigation across other VA systems is warranted in order to identify best practices as well as pitfalls of the e-consult mechanism. Additional work is needed to define what features of an e-consult program represent valid measures of access and quality care, and what monitoring systems, if any, need to be implemented.

## Abbreviations

e-consult: electronic consultation
EHR: electronic health record
PCPs: primary care providers
NP: nurse practitioner
VA: Veterans Affairs
VABHS: Veterans Affairs Boston Healthcare System
